# Occlusal Enamel Complexity in Middle Miocene to Holocene Equids (Equidae: Perissodactyla) of North America

**DOI:** 10.1371/journal.pone.0090184

**Published:** 2014-02-27

**Authors:** Nicholas A. Famoso, Edward Byrd Davis

**Affiliations:** Department of Geological Sciences and Museum of Natural and Cultural History, University of Oregon, Eugene, Oregon, United States of America; Monash University, Australia

## Abstract

Four groups of equids, “Anchitheriinae,” Merychippine-grade Equinae, Hipparionini, and Equini, coexisted in the middle Miocene, but only the Equini remains after 16 Myr of evolution and extinction. Each group is distinct in its occlusal enamel pattern. These patterns have been compared qualitatively but rarely quantitatively. The processes influencing the evolution of these occlusal patterns have not been thoroughly investigated with respect to phylogeny, tooth position, and climate through geologic time. We investigated Occlusal Enamel Index, a quantitative method for the analysis of the complexity of occlusal patterns. We used analyses of variance and an analysis of co-variance to test whether equid teeth increase resistive cutting area for food processing during mastication, as expressed in occlusal enamel complexity, in response to increased abrasion in their diet. Results suggest that occlusal enamel complexity was influenced by climate, phylogeny, and tooth position through time. Occlusal enamel complexity in middle Miocene to Modern horses increased as the animals experienced increased tooth abrasion and a cooling climate.

## Introduction

Horses have long been used as a primary example of evolution through adaptation to a changing environment [1], [2], [3]. Horse adaptations to changing climates, specifically through dental evolution in response to an increasingly abrasive diet, have been qualitatively analyzed, but rarely investigated quantitatively [4], [5], [6], [7]. Grass phytoliths have often been invoked as a primary driver of ungulate dental evolution [8], but recent work has suggested a much greater role for grit from drier environments and a reduced or even no role for phytoliths [9], [10],[11],[12]. Previous work on equid adaptation to an abrasive diet focused on changes in hypsodonty and enamel microstructure [8], [12], [13]. Evolution of horse teeth through an increase in hypsodonty, quantified as Hypsodonty Index (HI, the ratio of mesostyle crown height to occlusal length) [14], [15], [16], [17], [18], has been documented in the Oligocene through Pleistocene fossil record, primarily for North America [19]. Increased tooth height provides more resistive enamel over an animal’s lifetime. These changes have been interpreted as an adaptation to feeding in open habitats as cooling and drying climates changed woodlands to grasslands, requiring horses to adapt to increased rates of tooth wear created by environmental grit and the phytoliths of grasses [2], [8], [12]. Pfretzschner [13] investigated changes in equid enamel microstructure, concluding that adaptation to increased tooth wear was in place by the rise of “*Merychippus*” at about 19 Ma. The prisms and interprismatic matrix that make up enamel at the microscopic level stiffen enamel and the arrangement of these prisms strengthens it with respect to mechanical stress patterns from grinding against opposing teeth and food [13].

**Figure 1:**
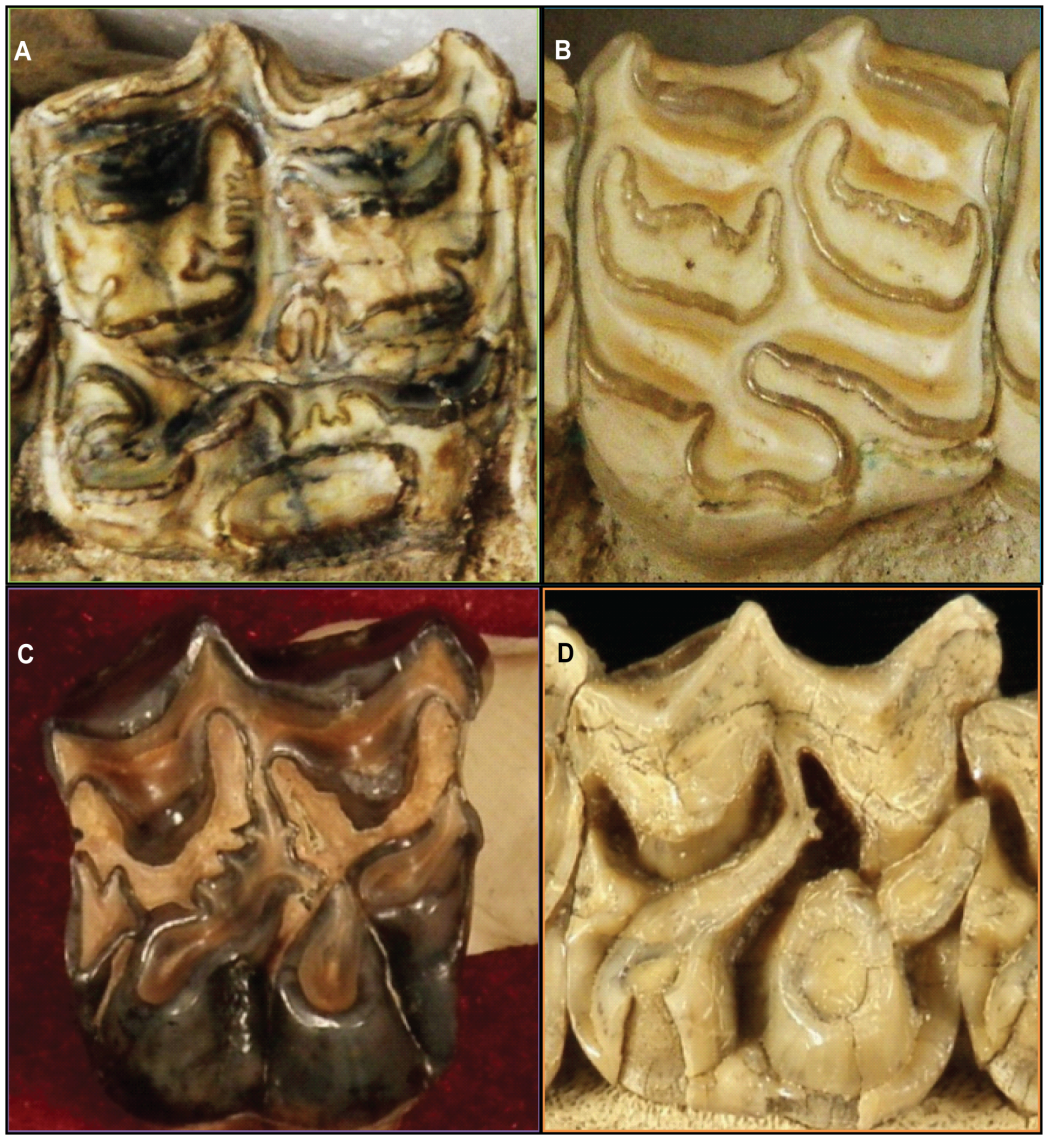
Representative teeth of each tribal-level group in this study. (A) Hipparionini, (B) Equini, (C) “Merychippini,” and (D) “Anchitheriini.” Each tribe has a distinct enamel pattern; the patterns decrease in complexity from A to D.

Miocene and later equid teeth are marked by complex, sinuous bands of enamel on their occlusal (chewing) surface (Fig. 1). These bands have taxonomically distinct patterns, with workers suggesting that members of the Equine tribe Hipparionini have more complex enamel bands than members of the tribe Equini [4], [5]. Previous workers have observed qualitatively that occlusal enamel increases in complexity over the evolutionary history of horses [5]. This change is suggestive because, in a similar way to increases in hypsodonty, increasing the occlusal enamel complexity of teeth should allow them to last longer simply by distributing lifetime tooth wear over a greater total resistive cutting area. Recent work has begun exploring the relationship between the complexity of ungulate occlusal enamel and abrasiveness of diet using quantitative methods [7], [20], [21], [22]. Here we assess the evolution of enamel complexity in Miocene and later North American equids in terms of occlusal enamel complexity, specifically investigating whether enamel complexity evolves in a pattern consistent with that expected as a response to increasing dietary abrasion. Additionally, we provide the first quantitative test of the relative complexity of hipparionine and equine occlusal enamel bands.

### Questions

Given current hypotheses of horse phylogeny and diversification in response to environmental changes and the extremely large available sample size (>2,581 known North American localities with fossil equids), we can use equid occlusal enamel band length and complexity of the occlusal surface to investigate the evolution of morphology in response to an increasingly abrasive diet. These observations lead to a series of questions: Do equids change their enamel complexity from the Miocene through the Recent? If so, does complexity increase over time, as would be expected for increasing adaptation to an abrasive diet? Is there a difference in enamel complexity between equid tribes, especially Hipparionini and Equini? If the evolution of enamel complexity is consistent with dietary adaptation, are there compromises between hypsodonty and enamel complexity? If so, do the two tribes make different compromises?

### Hypothesis

We hypothesize that increased abrasion in equid diets produced a selective advantage for teeth with greater resistive cutting area (occlusal enamel complexity).We will test this hypothesis by statistical analysis of enamel complexity derived from images of fossil horse teeth. If the statistical analysis shows a distinct pattern, then equids responded to increased abrasion through an increase in occlusal enamel complexity, providing an increased resistive cutting area for food processing during mastication. If the statistical analysis shows a pattern indistinguishable from random, we will be unable to reject the null hypothesis of no unifying adaptive significance to changes in occlusal enamel complexity or that some other process that we have not tested is controlling occlusal enamel complexity. Occlusal enamel complexity will vary as a consequence of phylogenetic constraint and evolutionary response to changes to ecological role through time. If our hypothesis is correct, the complexity of enamel on the occlusal surface of equid teeth should increase through time, tracking changes in the abrasiveness in diet as climates changed through the Neogene.

It is possible that phylogenetic constraint, inherited developmental or other limits to adaptation, may control the compromises different lineages of horses find between hypsodonty and enamel complexity for their adaptation to tooth abrasion. If so, we would expect each tribe to have distinct differences in their occlusal enamel complexity in comparison to their hypsodonty. Published qualitative observations of equid tooth morphology and its relationship to diet [7], [21], [22] suggest to us that Hipparionini should have the most complicated occlusal enamel, followed by Equini, then the “*Merychippus*” grade horses, and finally “Anchitheriinae”.

## Background

### Evolutionary Context

**Figure 2:**
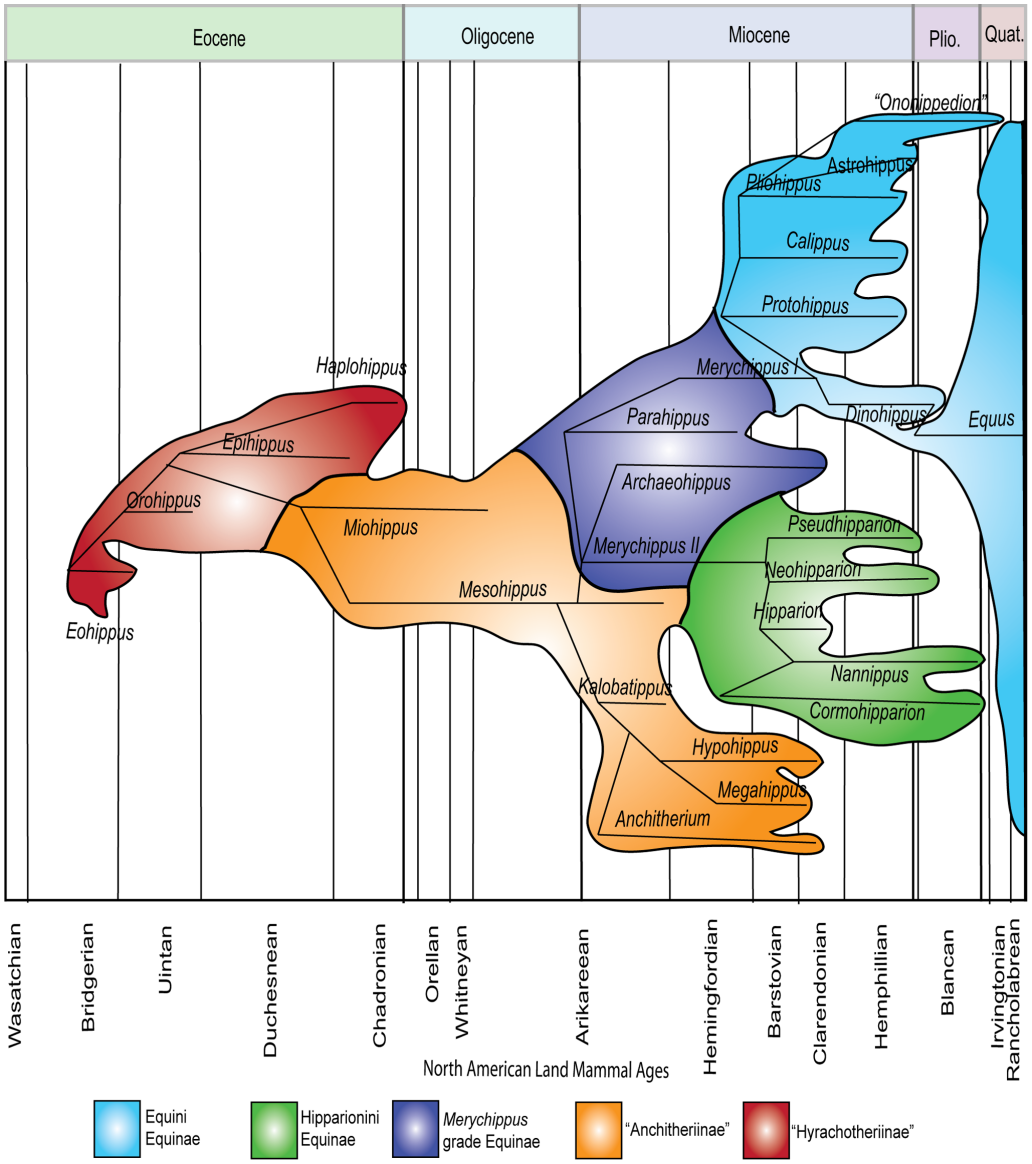
Phylogeny of Equidae used in this study (after MacFadden [25]). North American Land Mammal Ages indicated on the bottom. The size of the colored regions represents relative diversity among the groups. Horizontal lines represent time ranges of each genus or clade. This study begins with the Barstovian to capture the most advanced Equinae with derived enamel prismatic structure.

Analyses of evolutionary adaptations must be investigated within the context of phylogeny [23]. Linnean taxonomy is a hierarchical naming system that was originally created in a pre-Darwinian context to describe similarity amongst organisms. Like most natural systems, phylogenetic relationships are more complicated than the initial set of categories defined by man. The current consensus on equid phylogeny includes three subfamilies, “Hyracotheriinae,” “Anchitheriinae,” and Equinae [5], [24], [25] (Fig. 2). Within Equinae, there are two sub-clades, the tribes Hipparionini and Equini, and a basal grade mostly assigned to “*Merychippus*.” This genus has long been considered a paraphyletic taxon, maintained through convenience to include all basal equines that do not possess apomorphies of either Equini or Hipparionini. Typical “*Merychippus*” have an upper dentition that maintains the plesiomorphic features of the basal “Anchitheriinae,” a paraphyletic grade below Equinae (Fig. 1), but also share characters with derived Equinae [5], [26], [27]. Hipparionini and Equini have distinct tooth morphologies as well (Fig. 1). Members of the tribe Hipparionini are hypsodont, but relatively lower crowned and have more complicated enamel borders than their equin counterparts [4], [5], [24]. The two tribes of Miocene horses, Hipparionini and Equini, are diagnosed on the basis of differences of the structures formed by the folding of enamel on the occlusal surface of their teeth [4], [5], [6], [24], [25]. The shape of the occlusal pattern was shown to be an important character in equin and hipparionin phylogeny [5], [24], [28]. This qualitative difference leads us to ask whether complexity of occlusal enamel evolved differently because of phylogenetic constraint and/or climatic pressures between Equini and Hipparionini.

Because species are phylogenetically related to differing degrees, they cannot be considered as independent for statistical analysis [23]. To accommodate this dependence, Felsenstein [23] proposed the method of independent contrasts, incorporating the phylogenetic relationships into regression analysis. Independent contrasts has been developed into a broad field of phylogenetic comparative methods [29], [30], [31], but at this point all of them require phylogenies with branch lengths derived from models of molecular evolution. Ideally, we would use one of these comparative methods for testing our hypothesis of variations in the context of phylogeny, but current methods require known branch lengths and have yet to be adapted to fossil-based morphological phylogenies [32], [33], [34].

We will accommodate phylogenetic interdependence amongst the fossil horses by using nested variables in a multi-way analysis of variance (ANOVA). In this way, we are able to model phylogeny using the hierarchical taxonomic system as a proxy for phylogeny [7]. Using these nested variables in an ANOVA is not ideal for phylogeny, because it does not completely take the topology of a phylogenetic tree into account, but as a coarse approximation, it functions for this scale of analysis.

### Measures of Complexity

**Figure 3:**
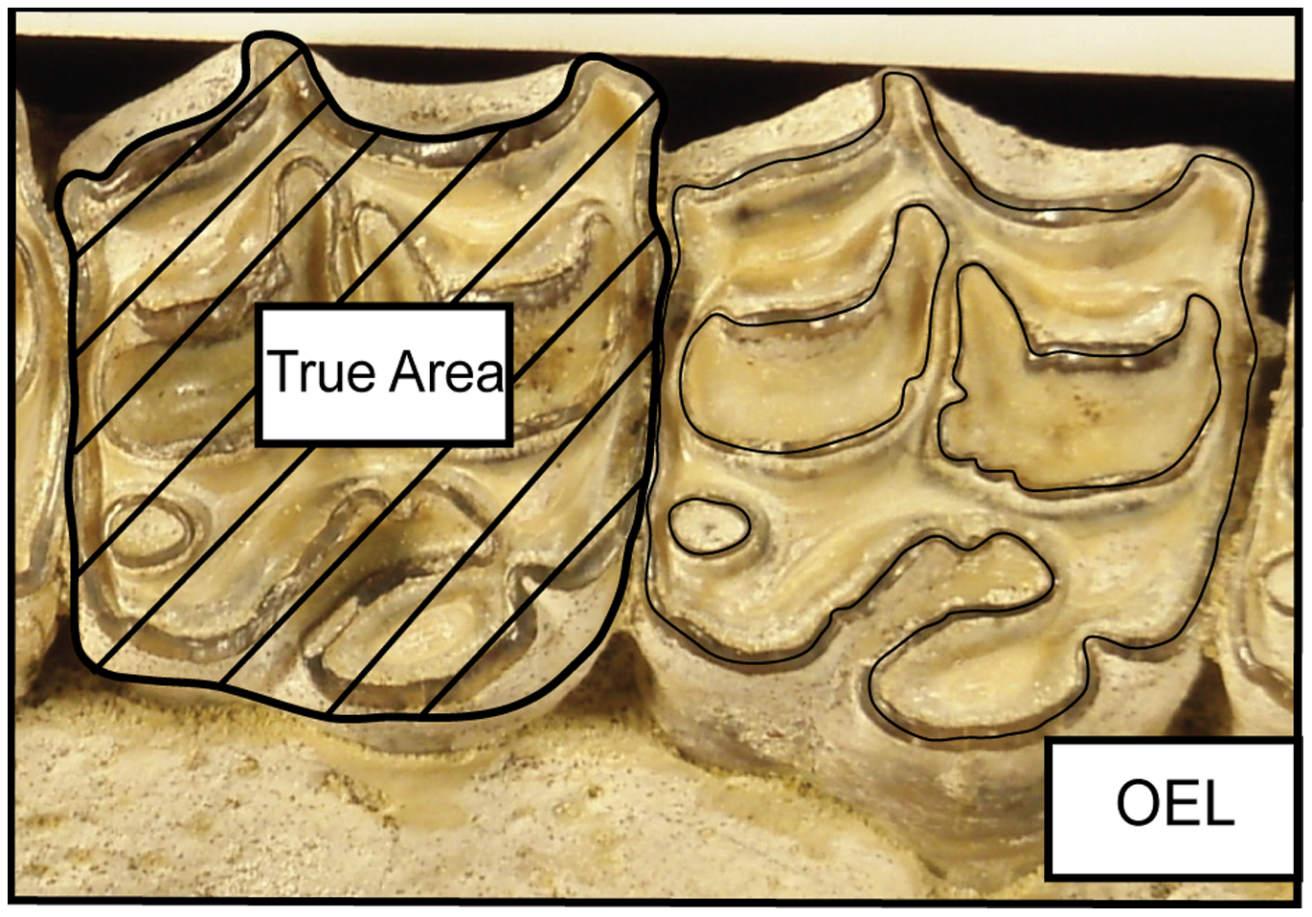
Examples of True Area and Occlusal Enamel Length (OEL) taken on digital image of *Pseudhipparion* sp. (UNSM 125531). True Area is a different measurement than length by width. These measurements are calculated with ImageJ. Figure is based on methodology presented by Famoso et[7].

Species and other higher taxonomic groups in horses are primarily diagnosed by qualitative characters; in fact, a majority of equid diagnoses rely upon differences in pattern of occlusal enamel [4], [24]. A complicated enamel pattern should have longer occlusal enamel length thus producing more enamel per unit surface area on the occlusal plane. Famoso et al. [7] introduced a numerical method to quantitatively measure and test the differences in enamel complexity in ungulates, a unit-less value called Occlusal Enamel Index (OEI): *OEI* = *OEL/√(True Area)* where *OEL* is the total length of enamel bands exposed on the occlusal surface as measured through the center of the enamel band, and *True Area* is the occlusal surface area constructed as a polygon following the outer edge of the occlusal surface, including any cementum that may exist outside of the enamel, where cementum on the lingual side is part of the occlusal surface while that on the buccal is not (Fig. 3). The True Area is not an occlusal length multiplied by width, but is instead representative of the area actually contained within the curved occlusal boundaries of the tooth. We are measuring True Area as a 2D projection, so we do not account for any increases in area that might arise from topography on the occlusal surface of the tooth. Because most equid teeth are on the low-relief end of the mesowear spectrum [34], this projection will have little effect on our current study; however, studies that extend this methodology to high-relief teeth might find improvements from a 3D approach. Analyzing images of teeth in the computer allows us to use the more precise true area instead of the more traditional technique of multiplying the measured length and width of the occlusal surface. True area is a proxy for body size, so OEI removes the effects of absolute scale on complexity; however, the effects of body size are not completely removed, as OEI does not adjust for size-related differences in complexity, i.e., allometry [7].

Becerra et al. [36] have introduced a similar enamel complexity metric, applying it to rodents. The enamel index (EI) is calculated as: *EI* = *OEL*/*(True Area)*. OEI differs from EI in that the occlusal area is treated differently. OEI produces a unitless metric while EI does not, producing values in units of 1/length, so consistent length scales would have to be used to maintain comparability among analyses. Becerra et al. [36] found evidence to suggest that selective pressures from regional habitats, in particular vegetation, have shaped the morphological characteristics of the dentition of caviomorph rodents in South America.

We use OEI for this study for three reasons: (1) we expect the unitless index to more completely account for isometric changes of enamel length with mass, (2) we want our results to be directly comparable to Famoso et al. [7], and (3) the unitless index is methodologically aligned to the unitless HI commonly used in horse paleoecology.

Two recent studies have analyzed enamel complexity within the Order Artiodactyla, using a slightly different approach that focuses more on visible enamel band orientation. Heywood [21] analyzed molar occlusal surfaces and characterized them on the basis of length, thickness, and shape of the enamel bands, concluding that plant toughness is a primary driver of occlusal enamel form in bovids. Kaiser et al. [22] investigated the arrangement of occlusal enamel bands in the molars of ruminants with respect to diet and phylogeny, finding that larger ruminants or those with higher grass content in their diet have a higher proportion of enamel ridges aligned at low angles to the direction of the chewing stroke.

Previous work on occlusal enamel patterns in equids has been limited to the observation that patterns change through wear stages [5], [37]. Famoso and Pagnac [6] suggested that the differences in occlusal enamel patterns through wear correspond to evolutionary relationships in Hipparionini. To date, attempts at quantifying the patterns of evolutionary change in occlusal enamel complexity between and within these equid tribes have been limited by small sample sizes [6], [7].

### Tooth Position

Beyond the pressures of the environment, differential expression by tooth position is another aspect of enamel band evolution that may be linked to phylogeny. Famoso et al. [7] demonstrated that enamel complexity is expressed significantly differently at each tooth position. Equid P2 and M3 are easily identifiable in isolation: the P2 has a mesially pointed occlusal surface while the M3 is tapered distally. The middle four teeth (P3-M2) are more difficult to identify to position when isolated as they have uniformly square occlusal surfaces. Premolars tend to be larger than molars within a single tooth-row, but size variation within a population overwhelms this difference for isolated teeth. As with many mammals, the majority of identifiable fossil equid material tends to be isolated teeth, as teeth are composed of highly resistant materials (enamel, dentine, and cementum) in comparison to the surrounding cranial bone. Many taxa, including *Protohippus placidus*, *Pliohippus cumminsii*, and *Hipparion gratum*, are only known from isolated teeth [1], [5], [24]. Because of their relative abundance in each tooth-row, a majority of isolated teeth tend to be the more difficult to distinguish P3 to M2. Including isolated teeth in our analysis would increase geographic and taxonomic diversity, but variation in enamel complexity amongst the tooth positions could overwhelm the signal. Optimizing the sample size in our study design makes it important to identify whether tooth position has a significant effect on OEI for P3 - M3.

## Methods

### Materials

Our data consists of scaled, oriented digital photographs of the occlusal surface of fossil and modern equid upper dentitions. We measured a total of 800 teeth from a broad selection of North American equids ranging from 16 Ma to recent (Table S1). Photographs were taken with Kodak DC290, Fujifilm Finepix A345, Olympus Stylus Tough, and Canon Digital EOS Rebel SLR cameras. Some data were collected from Famoso et al. [7]. Some images were used with permission from the UCMP online catalog (http://ucmpdb.berkeley.edu/). Specimen numbers and repository information are reported in Table S1, and geographic locations of repositories are indicated in the Institutional Abbreviations section. Each named museum listed in the Institutional Abbreviations section gave us permission to access their collections. Care was taken to select individuals in medial stages of wear (no deciduous premolars and no teeth in extreme stages of wear). Skulls and complete to nearly complete tooth rows were preferred because we can be more confident in taxonomic identification and tooth position. Isolated teeth were also included when more complete tooth-rows were not available for a taxon.

### Institutional Abbreviations

**UNSM** = University of Nebraska State Museum, Lincoln, NE; **UOMNCH** = University of Oregon Museum of Natural and Cultural History, Eugene, OR; **UCMP** = University of California Museum of Paleontology, Berkeley, CA; **MVZ** = University of California Museum of Vertebrate Zoology, Berkeley, CA; **AMNH F:AM** = Frick Collection, American Museum of Natural History, New York, NY; **AMNH FM** = American Museum of Natural History, New York, NY; **UF** = University of Florida Museum of Natural History, Gainesville, FL; **JODA** = John Day Fossil Beds National Monument, Kimberly, OR; **CIT** = California Institute of Technology (Cast at JODA); **UWBM** = University of Washington Burke Museum of Natural History and Culture, Seattle, WA; **SDSM** = South Dakota School of Mines and Technology Museum of Geology, Rapid City, SD; **USNM** = United States National Museum of Natural History, Washington, DC.

### Occlusal Enamel Index

Enamel length and True Area of each tooth were measured using the NIH image analysis program ImageJ (http://rsb.info.nih.gov/ij/). Site geology (formation and member), time period (epoch and North American Land Mammal Age [NALMA]), tooth position (if known), physiographic region, political region, and taxonomy (subfamily, tribe, genus, and species) were recorded for each specimen. Data were stored in a Microsoft Excel 2010 spreadsheet (Table S1). OEI was calculated following Famoso et al. [7] (Fig. 3).

We used one-way and multi-way analysis of variances (ANOVAs) in JMP Pro 9 to determine whether the relationship between tooth size and enamel length fit our predictions. We used a Shapiro-Wilk W test [38] to test whether OEI values were normally distributed and the Bartlett test of homogeneity [39] to determine whether the variances in OEI among groups were homogeneous. If OEI is normally distributed and the variances are homogeneous among groups, then the data will not violate the assumptions of the ANOVA and a parametric test can be performed. ANOVA is generally robust to violations of both of these assumptions, particularly if the sample sizes amongst groups are similar [40]. Our sample sizes are not similar among all of our groups, so we have supplemented ANOVAs with nonparametric Wilcoxon tests [41] when one or both of these assumptions are violated. When data from all tooth positions were pooled, they did not display a normal distribution. Upon further investigation, we determined that all but one position in the tooth row was normally distributed and excluded the non-normal tooth (M3) from further analysis. As discussed below, we used nested (hierarchical) ANOVAs to account for evolutionary relatedness in our analysis. Nested ANOVAs include levels of independent factors which occur in combination with levels of other independent factors. Because ANOVAs can only provide a test of all factors together, we have included Tukey-Kramer tests where needed to investigate statistically significant groupings [40].

An analysis of tooth position was run on a subset of the data (n = 528 teeth) with known tooth position. This ANOVA allowed us to determine whether there was a tooth position or group of tooth positions with indistinguishable OEI values, allowing us to limit the number of specimens to be measured for the subsequent analyses. The results of this analysis would provide a justification for the selection of a subset of teeth to consistently measure. We ran a multi-way ANOVA with OEI as the dependent variable and tribe, region, NALMA, and tooth position as the independent factors. P2 and M3 were excluded as they have an overall different shape and are statistically different in OEI from the teeth from the middle of the tooth-row [7]. We additionally ran a one-way ANOVA with OEI as the dependent variable and tooth position excluding P2 and M3 as the independent factor. Tukey-Kramer tests [42] were also performed to investigate the origin of significance for independent factors. We also ran a one-way ANOVA with OEI as the dependent variable and tooth position excluding P2 and M3 for the subset of the data that only belonged to the genus *Equus*, the genus with the largest overall sample size. Using just one genus would eliminate any influence from higher level evolutionary relationships. A one-way ANOVA with OEI as the dependent variable and tooth position excluding P2 and M3 by tribe (just Equini, just Hipparionini, and just “Anchitheriinae”) allowed us to test whether variation in tooth position was consistent at this level of lineage. Tribal affiliations were used as a proxy for phylogenetic relationships, therefore all genera needed a tribal level affiliation to be included in the ANOVAs, but the basal members of the Equinae (members of the “*Merychippus*” grade) do not belong to the Hipparionini or Equini, so we applied the place-holder paraphyletic tribe “Merychippini.” Similarly, for all members of the paraphyletic subfamily “Anchitheriinae,” the place-holder name “Anchitheriini” was applied.

Running our analyses above the genus level limits the influence of lumping and splitting at the genus and species levels which arise from qualitative analysis of characters found in isolated elements. While working through museum collections, we found several *nomen nudum* manuscript names assigned to specimens. We assigned these specimens to the most appropriate, currently-established genus name and left the species as indeterminate. Even for published species of equids, there are ongoing controversies about the validity of names. Major problem areas include genera and species split from the paraphyletic form genus “*Merychippus*” [5], [43], [44], [45], [46] as well as the number and identity of Plio-Pleistocene and recent *Equus* species [5], [42], [47], [48]. There has been controversy as to the validity of the number of genera and species that belong to Hipparionini [5], [15], [37], [43], [45], [49], [50]. Leaving the analysis above the genus level removes any effect taxonomic uncertainty at the generic and specific levels.

Limiting the taxonomy to the Tribe and above also allows a more robust sample size. Equid genera are typically diagnosed through a combination of dental and cranial characters [5], [24], [51], [52]. Most isolated dental specimens can only be identified to genus because of the lack of diagnostic features, so a genus or tribal cutoff for our analysis allows us to access the rich supply of isolated teeth.

It was necessary to combine two of the NALMAs, the Irvingtonian and Rancholabrean, to have sufficient sample size for the analyses used here. This combination is not ideal as it eliminates a portion of the temporal resolution of our study. The Irvingtonian and Rancholabrean are both part of the Pleistocene. The Irvingtonian was not well sampled enough to analyze on its own, and by combining it with the Rancholabrean we were also able to include specimens from the Pleistocene in the temporal bin when their NALMA was not known.

To accurately investigate OEI through hierarchical taxonomic relationships and changing regions through time, it was necessary to use nested terms in our analyses. Nesting tests hypotheses about differences among samples which are placed in hierarchical groups. Nested factors are usually random-effects factors, or a factor with multiple levels but only a random sample of levels is included in the analysis. When applied to an ANOVA, it is considered a modified one-way ANOVA [40] where one variable is the random-effects factor and the other is considered a subsample. Including nested factors accounts for within-group variability.

To make a single overall test of our hypothesis, we constructed a multi-way ANOVA with OEI as the dependent variable and tooth position, nested taxonomy (tribe within subfamily), and time (NALMA) as independent factors (Listed in the Results section as Nested Multi-way Analysis of Variance). In addition, we ran three groups of one-way ANOVAs with Tukey-Kramer tests to test our hypothesis of the influence of climate and phylogeny as on OEI through time. Our one-way ANOVAs use OEI as the dependent variable. Our first group of one-way ANOVAs (in Results as ANOVA 1: OEI vs. Tribe) uses tribe as the independent variable to investigate how OEI differs between lineages. Next, we used NALMA as the independent variable to examine how overall OEI changes through time (ANOVA 2: OEI vs. NALMA). Finally, we used tribe as the independent variable and separated by NALMA to explore whether the different lineages are distinct in OEI at different periods of time (ANOVA 3: OEI vs. Tribe within Each NALMA).

## Results

All datasets were tested for the assumptions of ANOVA, Gaussian distribution and equality of variances among groups. For concision, only significant violations of these assumptions are noted.

### Tooth Position

The Bartlett test of equal variance for this ANOVA showed significant differences among variances for this subset of the data, so we supplemented the standard ANOVA with a Wilcoxon test for the comparison (Table 1). The Chi Square approximation of the Wilcoxon was not significant (*p* = 0.0757), in contrast to the one-way ANOVA with OEI as the dependent variable and tooth position (excluding P2 and M3), which was significant at *p = *0.0124. The Tukey-Kramer test indicates that P3, P4, and M2 are not significantly different from one another and P3, M1, and M2 are not significantly different from one another (Table 2). The P4 and M1 appear to be significantly different from each other, but recall that the Tukey-Kramer test relies upon the pooled variances of the ANOVA. The Bartlett test of equal variances was not significant for the one-way ANOVA with OEI as the dependent variable and tooth position (excluding P2 and M3) for *Equus*. The Tukey test for *Equus* shows a slightly different pattern from the overall data set, P4 and M1 are significantly different from each other, P3 overlaps with P4 and M2, and M2 overlaps with P3 and M1. The Bartlett test of equal variance for the one-way ANOVAs with OEI (dependent variable) and tooth position (excluding P2 and M3) was not significant for Equini, but was significant for Hipparionini. The ANOVA for Equini was not significant, showing no significant differences among tooth positions. The Chi Square approximation of the Hipparionini Wilcoxon test was not significant (*p = *0.3334). The Hipparionini ANOVA was also not significant (*p = *0.0687) and the Tukey test showed no significant differences among tooth positions.

**Table 1:** Results of Tooth Position Wilcoxon Test.

Level	Count	ScoreSum	ExpectedScore	ScoreMean	(Mean-Mean0)/Std0
M1	70	8707	10010	124.386	−2.175
M2	72	10655	10296	147.986	0.593
P3	68	9454	9724	139.029	−0.454
P4	75	11939	10725	159.187	1.981

**Table 2:** Results of Tooth Position ANOVA and Tukey-Kramer Test.

Tooth Position	Group	Mean OEI
P4	A	17.676
M2	AB	16.415
P3	AB	16.357
M1	B	15.857

### Nested Multi-way Analysis of Variance

All independent variables are significant for OEI at the α = 0.05 level. Table 3 shows the *p* values for each variable.

**Table 3:** Results of the Nested Multi-way ANOVA.

Dependent Variable	NALMA	Tooth Position	Subfamily	Tribe [Subfamily]
OEI	*p*<0.0001	*p*<0.0001	*p*<0.0001	*p*<0.0001
*F* test value	0.310	0.027	0.080	0.139

### ANOVA 1: OEI vs. Tribe

The Bartlett test of equal variance for this ANOVA was significant, so we supplemented the standard ANOVA with a Wilcoxon test for the comparison (Table 4). The Chi Square approximation of the Wilcoxon was significant (*p*<0.0001), matching the ANOVA results (*p*<0.0001) (Table 5). Tukey test results indicate that Hipparionini and Equini are separate from one another. “Merychippini” is between the Hipparionini and Equini. The “Anchitheriini” is in its own distinct group.

**Table 4:** Results of Wilcoxon test for OEI vs Tribe.

Level	Count	Score Sum	Expected Score	Score Mean	(Mean-Mean0)/Std0
Anchitheriini	36	4820	11574	133.889	−6.246
Merychippini	45	16014.5	14467.5	355.878	1.289
Equini	375	115304	120563	307.476	−2.27
Hipparionini	186	70265	59799	377.769	4.909

**Table 5:** Results of ANOVA and Tukey-Kramer Test for OEI vs Tribe.

Tribe	n	Group	Mean OEI
Hipparionini	186	A	10.026
“Merychippini”	45	AB	9.903
Equini	375	B	9.602
“Anchitheriini”	36	C	8.350

### ANOVA 2: OEI vs. NALMA

The Bartlett test of equal variance for this ANOVA was significant, so we supplemented the standard ANOVA with a Wilcoxon test for the comparison. Results are presented in Table 6. The Chi Square approximation was significant (*p*<0.0001). The Wilcoxon test yields similar results to the standard ANOVA (Table 7), which was also significant (*p*<0.0001). The Irvingtonian/Rancholabrean stands out as a unique period of time with the highest OEI values. The Blancan has the next highest OEI values. The Recent is grouped alone with the lowest OEI values. The Clarendonian, Hemphillian, and Barstovian overlap with the Blancan and the Recent and have OEI values that are intermediate between the two groups.

**Table 6:** Results of Wilcoxon tests for OEI vs NALMA.

Level	Count	Score Sum	Expected Score	Score Mean	(Mean-Mean0)/Std0
Barstovian	147	39029	46525.5	265.503	−3.865
Blancan	67	22358	21205.5	333.701	0.815
Clarendonian	156	46218.5	49374	296.272	−1.594
Hemphillian	96	25101.5	30384	261.474	−3.206
Irv/Rancho	126	59166	39879	469.571	10.517
Recent	40	8155	12660	203.875	−4.03

**Table 7:** Results of ANOVAs and Tukey-Kramer Tests for OEI vs NALMA.

Level	Group	Mean OEI
Irv/Rancho	A	10.799
Blancan	B	9.790
Clarendonian	BC	9.504
Hemphillian	BC	9.301
Barstovian	BC	9.300
Recent	C	8.957

### ANOVA 3: OEI vs. Tribe within Each NALMA

The Bartlett test for the Hemphillian ANOVA was significant, so we supplemented the standard ANOVA with a Wilcoxon test for that interval (Table 8. All tests for NALMAs were significant (Table 9). The Barstovian (*p*<0.0001) had two statistical groupings; one group is the “Merychippini” and Hipparionini, and the other is the Equini and “Anchitheriini.” The Clarendonian (*p*<0.0001) had the same two groups. The Hemphillian (*p*<0.0001) and the Blancan (*p = *0.0013) both have two distinct groups, the Hipparionini and Equini. The groupings of tribes stay the same through time.

**Table 8:** Results of Wilcoxon test for OEI vs Tribe within each NALMA.

Hemphillian					
Level	Count	ScoreSum	ExpectedScore	ScoreMean	(Mean−Mean0)/Std0
Equini	53	1929.5	2570.5	36.406	−4.719
Hipparionini	43	2726.5	2085.5	63.407	4.719

**Table 9:** Results of ANOVAs and Tukey-Kramer Tests for OEI vs Tribe within each NALMA.

NALMA	Tribe	N	Group	Mean OEI
Barstovian	Hipparionini	35	A	9.927
Barstovian	“Merychippini”	38	A	9.897
Barstovian	Equini	43	B	8.868
Barstovian	“Anchitheriini”	30	B	8.453
Clarendonian	“Merychippini”	5	A	10.31
Clarendonian	Hipparionini	95	A	9.969
Clarendonian	Equini	52	B	8.712
Clarendonian	“Anchitheriini”	4	B	7.771
Hemphillian	Hipparionini	43	A	10.028
Hemphillian	Equini	53	B	8.711
Blancan	Hipparionini	7	A	10.857
Blancan	Equini	60	B	9.666

## Discussion

Tooth position does not significantly affect OEI for the middle four teeth (P3- M2) of the upper tooth row at the tribal level. Our investigation into tooth position indicates that we can safely include isolated molariform teeth in our study without taking tooth position into account if we exclude the P2 and M3. These two teeth have already been shown to be different from the other molariform teeth [7]. We also found that our data were normally distributed when the P2 and M3 were excluded. We found more variation in OEI for the P4 than for the M1, M2, and P3 according to Bartlett’s test. We suggest subsequent work should focus on M1, M2, or P3 to take advantage of this lower variance. It is important to note that the Wilcoxon test was not significant for the main body of the data. While the ANOVA was significant, this dataset violated the assumption of equal variance, so the Wilcoxon is the more appropriate test. In the end, all of our analyses of tooth position suggest that the middle four teeth are not significantly different from one another and can be used interchangeably in an analysis at this broad a level. Our investigation into tooth position also explored whether the variation in OEI for the various tooth positions were the same among horse lineages. Within each tribe tooth position is not significant for the four square middle teeth. Tooth position OEI varies significantly between tribes, suggesting that each lineage is adapting differently for each tooth.

The results of our nested multi-way ANOVA indicate that time, tooth position, and nested taxonomy are all significant factors for the length of enamel in horse teeth. Each of the subsequent one-way ANOVAs allowed us to tease apart the details of the multi-way ANOVA result.

Generally, OEI increases from the Miocene NALMAs to the Pleistocene, correlating with the overall cooling climate from the mid-Miocene Climactic Optimum (16 Ma) to recent [53]. This increase in OEI over time is compatible with our hypothesis that, as climate became cooler and dryer and the abrasiveness of the equid diet increased [12], increased OEI was selected for across horse lineages. OEI in the late Miocene is lower than in the Pliocene and the increase continues through to the Pleistocene. In the Holocene, we see a decrease in complexity to levels similar to that of the late Miocene. Increase in OEI though time matches the documented increase in HI through time [35]. OEI and HI are measures of ways in which ungulates increase the amount of enamel available for a lifetime of chewing abrasive foodstuffs, so higher values of either metric could suggest higher abrasiveness in diet [7].

The drop in complexity we observe in our Holocene sample could be influenced by the limitation in taxonomic sampling available for extant Equini. The only animals available for inclusion are influenced by conditions of artificial selection and human management, and are descended from the Old World lineage of horses unlike the New World fossils included in our dataset. These animals do not have the same diet, behavior, or morphology as they would in the wild [54], [55], so if enamel complexity is phenotypically plastic and reflects diet during tooth development, as suggested for elephantids and rodents [56], [57], [58], their simpler enamel may reflect the dietary conditions under domestication. This possibility warrants further investigation but is beyond the scope of our study. More likely is that the domestic and feral horses in our Holocene dataset are descended from animals in distinctly different selective regimes in the Old World; future studies with larger spatial sampling would be needed to test this hypothesis. At this point, we do not feel confident interpreting the drop in OEI from the Pleistocene to the Recent as an evolutionary change, but instead interpret it as suggestive of the biogeography of this trait.

The overall analysis of OEI by tribes (ANOVA 1) strongly supports the hypothesis that the Equini and Hipparionini had distinct evolutionary responses in occlusal enamel evolution. The results of the Tukey-Kramer test very closely reflect the evolutionary relationships of the family. Hipparionini and Equini are sister taxa, and both are in distinct groups from one another. “Merychippini” includes the common ancestor between these two within the subfamily and is grouped with both the Hipparionini and Equini as is expected in light of the phylogeny. “Anchitheriini” is the paraphyletic stem group ancestral to “Merychippini.” The “Anchitheriini” is in its own group statistically and has the lowest OEI. Members of “Anchitheriini” are low-crowned, or have low HI [5], and can be interpreted as either browsers or intermediate feeders with a low percentage of abrasive material in their diet. Browse comprises a larger portion of the diet for “Anchitheriini” than any of the other tribes, and if diet is shaping occlusal enamel evolution, this group should have the lowest OEI, as indeed it does. We can use geography to tease apart diet and environmental change. Incorporating independent diet proxies (e.g., stable isotopes from enamel and/or microwear) combined with a regional biogeographic approach in a future study would identify the relative impact of local environmental change versus changing diets in shaping the evolution of OEI.

ANOVAs for tribes by NALMAs present an interesting pattern that enhances our interpretation of occlusal enamel evolution in horses. Ancestry seems to be an important influence on enamel length: the characteristic OEI values for a group are established at its origin and persist through time. When we consider interpretations of diet for each group [25] we find an unexpected pattern: many of the Barstovian equin horses are interpreted to be grazers but have OEI values consistent with contemporaneous browsing taxa. Interestingly, HI values for these equin horses are higher than those of hipparionin horses, while the converse is true for OEI. This supports our qualitative assertion that equin horses have more hypsodont yet less complicated teeth then their hipparionin relatives.

When the four tribes are present, Hipparionini and “Merychippini” are grouped together. Equini and “Anchitheriini” are also grouped. This pattern is only seen in the Barstovian and Clarendonian. Groupings may either represent tribes closely competing for resources or more evidence for the importance of phylogenetic constraint in this character. That is, the sample for “Merychippini” may be dominated by ancestral forms of Hipparionini, producing the observed connection between the tribes. We suspect this may be the case because more of the equin “*Merychippus*” have been split out into their own genera [5], [43], [44], [45], [46].

Conversely, it is possible that typical fossil members of Equini were more intermediate feeders and competing with browsing “Anchitheriini” for resources. Notably, in Great Plains Clarendonian faunas, Equini and “Anchitheriini” both compose a small percentage of the relative abundance of horses [6]. The similarity in OEI and relative abundance between these two groups warrants further investigation because previous workers have assigned the equines to grazing niches on the basis of their hypsodonty and isotopic data [25], but their OEI values would suggest that they are browsing along with their contemporaneous anchitherine relatives.

In terms of species richness, Hipparionini were the most successful tribe during the Clarendonian in the Great Plains, but were eventually replaced by Equini at the end of the Blancan. “Anchitheriini” and “Merychippini” go extinct by the Hemphillian, leaving Equini and Hipparionini (Fig. 2). The two tribes are significantly different in the Hemphillian and Blancan. Hipparionini are constrained to the southern latitudes during the Blancan and are extinct by the end of the Blancan [59]. Hipparionini remain in regions closer to the equator where the effects of climate change would not have been as strong [53], [60]. In those regions, they continue to have higher OEI than their equin counterparts. The food source for hipparionines may have been restricted to warmer climates as the globe cooled, thus restricting the range of the tribe. The warmer regions may have served as refugia for North American hipparionin horses. We can better understand the drivers of occlusal enamel complexity when we can look across geography because we can compare regional patterns unfolding under slightly different environmental changes. Adding these data would allow us to investigate changes in response to regional climate changes through time.

We would like to apply these methods to other megafauna which have adaptations to increased ingested abrasiveness, such as camels, rhinos, African large primates, and South American notoungulates. A majority of enamel complexity in Equids is found in the hypsodont forms which originate in the Barstovian and are included in this study. However, it would be interesting to extend our methods back deeper into the Anchintheriinae and perhaps include the *Eohippus*-grade equids to see whether they also reflect other metrics of changing ecology. We would also like to test differences within Plio-Pleistocene *Equus* (e.g., caballine and stilt-legged horses), comparing them to Hipparionini genera to see if any equin horses are independently evolving complex enamel patterns similar to hipparionin horses as or after those hipparionins go extinct. This way we could test whether these Equini species converged on vacated niche space left by the extinct hipparionines.

The results of our Occlusal Enamel Index (OEI) study suggest that the complexity of the occlusal enamel of equid teeth is influenced by a combination of evolutionary relatedness, developmental constraint (tooth position), and changing environments over time. Equini seem to have an overall lower OEI than Hipparionini which supports the qualitative hypothesis that Equini have less occlusal enamel than Hipparionini. Our study shows that enamel band shapes are being influenced by climate and evolutionary history. As climate dries through time, we see an overall increase in enamel complexity. Phylogenetic relationships also have an influence on relative enamel complexity between clades (i.e., Equini tends to have less complex enamel than Hipparionini). Our results are consistent with the hypothesis that horses increase their enamel complexity in response to increased tooth abrasion from the Miocene through the Holocene.

## Supporting Information

Table S1 **Raw OEI data for statistical analysis.**
(XLSX)
